# Poster Session II - A254 DISCORDANCE BETWEEN BMI AND VISCERAL ADIPOSITY IDENTIFIES DISTINCT INFLAMMATORY PHENOTYPES IN INFLAMMATORY BOWEL DISEASES

**DOI:** 10.1093/jcag/gwaf042.254

**Published:** 2026-02-13

**Authors:** G Simkin, B Maracle, D Hazra, K Novak, C Lu, G G Kaplan, M Raman, J Besney, R Reji, A AlDarwish, C Seow, R Ingram, C Ma, R Panaccione, J St-Pierre

**Affiliations:** Medicine, University of Calgary, Calgary, AB, Canada; Medicine, University of Calgary, Calgary, AB, Canada; University of Calgary, Calgary, AB, Canada; Medicine, University of Calgary, Calgary, AB, Canada; Medicine, University of Calgary, Calgary, AB, Canada; Medicine, University of Calgary, Calgary, AB, Canada; Medicine, University of Calgary, Calgary, AB, Canada; Medicine, University of Calgary, Calgary, AB, Canada; Division of Gastroenterology and Hepatology, University of Calgary, Calgary, AB, Canada; Medicine, University of Calgary, Calgary, AB, Canada; Medicine, University of Calgary, Calgary, AB, Canada; Medicine, University of Calgary, Calgary, AB, Canada; Medicine, University of Calgary, Calgary, AB, Canada; Medicine, University of Calgary, Calgary, AB, Canada; Medicine, University of Calgary, Calgary, AB, Canada

## Abstract

**Background:**

Inflammatory bowel disease (IBD) is a chronic immune-mediated disorder with distinct metabolic consequences, including altered adipose distribution. Body mass index (BMI) estimates body composition but poorly reflects adipose distribution. Visceral adipose tissue (VAT) may capture metabolic and inflammatory risk more adequately, and can be conveniently measured by intestinal ultrasound (IUS).

**Aims:**

We aimed to define VAT-BMI phenotypes and assess their relationship with disease activity.

**Methods:**

Adult IBD patients undergoing IUS were stratified by BMI (<25 vs ≥ 25 kg/m2) and sex-specific median VAT thickness, yielding four VAT-BMI phenotypes: lean (low BMI/low VAT), visceral-predominant (low BMI/high VAT), peripheral (high BMI/low VAT), and combined (high BMI/high VAT). Disease activity, metabolic comorbidities, and biomarkers were compared across phenotypes. Composite disease activity was defined as active inflammation on ≥ 1 of the following within 3 months: IUS (BWT >3mm and/or hyperemia), endoscopic activity, CRP >8mg/L, fecal calprotectin >250mcg/g, or symptomatic activity. Continuous variables were analyzed using one-way ANOVA with Tukey’s post-hoc test for multiple comparisons when overall significance was achieved and categorical variables were analyzed using the Freeman-Halton test.

**Results:**

Among 124 participants (75 Crohn’s disease, 49 ulcerative colitis; 59% male), the median VAT thickness was 33.7mm in males and 28.6mm in females. Phenotype distribution was: lean 34%, peripheral 17%, visceral-predominant 13%, and combined 36%. Composite disease activity differed across phenotypes at 1 month (p = 0.0336): lean 71%, peripheral 52%, visceral-predominant 38%, and combined 44%. Findings were consistent at 3 months (p = 0.0028): lean 81%, peripheral 67%, visceral-predominant 38%, and combined 49%. IUS activity also varied significantly (p = 0.0004), with highest activity in lean and lowest in combined/visceral-predominant phenotypes. Total cholesterol was higher in combined cases (p = 0.049). Diabetes and hypertension were more frequent in the combined and peripheral phenotypes, though not statistically significant (both p = 0.088), likely due to small sample size.

**Conclusions:**

VAT-BMI discordance identifies distinct inflammatory and metabolic phenotypes in IBD. Lower visceral adiposity was associated with greater inflammation, suggesting a potential protective role of VAT. Incorporating VAT assessment may enhance disease phenotyping and risk stratification.

A254 Table 1: Distribution of VAT-BMI Phenotypes and Corresponding Disease Activity in IBD

**Funding Agencies:**

Dr Keith MacCannell Rising Researcher Scholar Award Endowment

**Table:** 

	Low VAT	High VAT
BMI <25	Lean (n = 42)	Visceral-predominant (n = 16)
	IUS active: 43%	IUS active: 13%
	Composite active: 81%	Composite active: 38%
BMI >25	Peripheral adiposity (n = 21)	Combined (n = 45)
	IUS active: 24%	IUS active: 4%
	Composite active: 67%	Composite active: 49%

